# Simultaneous and Continuous Estimation of Shoulder and Elbow Kinematics from Surface EMG Signals

**DOI:** 10.3389/fnins.2017.00280

**Published:** 2017-05-30

**Authors:** Qin Zhang, Runfeng Liu, Wenbin Chen, Caihua Xiong

**Affiliations:** The State Key Laboratory of Digital Manufacturing Equipment and Technology, Institute of Rehabilitation and Medical Robotics, Huazhong University of Science and TechnologyWuhan, China

**Keywords:** principle component analysis (PCA), independent component analysis (ICA), artificial neural network (ANN), simultaneous and continuous motion estimation, myoelectric control

## Abstract

In this paper, we present a simultaneous and continuous kinematics estimation method for multiple DoFs across shoulder and elbow joint. Although simultaneous and continuous kinematics estimation from surface electromyography (EMG) is a feasible way to achieve natural and intuitive human-machine interaction, few works investigated multi-DoF estimation across the significant joints of upper limb, shoulder and elbow joints. This paper evaluates the feasibility to estimate 4-DoF kinematics at shoulder and elbow during coordinated arm movements. Considering the potential applications of this method in exoskeleton, prosthetics and other arm rehabilitation techniques, the estimation performance is presented with different muscle activity decomposition and learning strategies. Principle component analysis (PCA) and independent component analysis (ICA) are respectively employed for EMG mode decomposition with artificial neural network (ANN) for learning the electromechanical association. Four joint angles across shoulder and elbow are simultaneously and continuously estimated from EMG in four coordinated arm movements. By using ICA (PCA) and single ANN, the average estimation accuracy 91.12% (90.23%) is obtained in 70-s intra-cross validation and 87.00% (86.30%) is obtained in 2-min inter-cross validation. This result suggests it is feasible and effective to use ICA (PCA) with single ANN for multi-joint kinematics estimation in variant application conditions.

## 1. Introduction

Surface Electromyography (EMG) signal represents ample electric information of muscle contractions innervated by neural signals. It has been used as a viable and effective neural interface in interpreting human's movement intent into control command especially for various robotic systems (Farina et al., 2014; Maciejasz et al., 2014). Traditionally, the predefined motion patterns are classified from EMG features through the trained motion classifier (Kuiken et al., 2009; Chen et al., 2013; Ju and Liu, 2014). As long as a predefined motion pattern is selected, the robot will be driven along a predefined trajectory although the user would like change their movement intent. For multi-DoF coordinated motions, motion patterns of each DoF are respectively classified and sequentially employed to drive the robot to achieve the intended coordinated movement. Such motion decoding style based on pattern-classification technology is different from the natural neuromuscular control strategy where the human's intent (represented by EMG) is consecutively mapped to the human's motion (Farina et al., 2014). Therefore, simultaneous and continuous motion estimation has been proposed as an alternative manner to achieve natural and intuitive motor control.

Comparing with the motion pattern classification, the simultaneous and continuous motion estimation provides concurrent motion information (such as joint angle, muscle force) rather than motion trigger command together with the EMG recording. With simultaneous and continuous motion intent decoding method, the estimates of the motion information can be simultaneously obtained which is more efficient especially for coordinated motion estimation with multiple DoFs involved. This procedure continuously proceeds with the persisted recording and interpreting of the EMG signals. Although the continuous motion estimation of one or two DoFs at one joint (such as elbow Zhang et al., 2013; Han et al., 2015, wrist and/or hand Jiang et al., 2012; Muceli and Farina, 2012 or finger Ngeo et al., 2014; Pan et al., 2014) has drawn a lot of attention, there is few research on continuous estimation of multiple DoFs across shoulder and elbow joint (Au and Kirsch, 2000; Artemiadis and Kyriakopoulos, 2010, 2011). Thus, the prosthetics (Miller et al., 2008), exoskeletons (Kiguchi et al., 2008; Kiguchi and Hayashi, 2012) and other assistive devices for upper limb rehabilitation (Vogel et al., 2013; Jang et al., 2016) cannot benefit from the continuous motion estimation technology. This paper investigates the feasibility of the simultaneous and continuous estimation of 4-DoF kinematics across shoulder and elbow from EMG signals during coordinated arm movements.

Due to the nonlinearity of the mapping between the EMG and the human motions as well as the non-stationarity of the EMG signals over time, the performance of myoelectrical control heavily depends on the accuracy and robustness of the human motion estimation. To achieve this purpose, various research efforts were placed on exploring the correlation between EMG and kinematics or dynamics during different physiological or application conditions as well as developing feasible and effective EMG feature extraction and estimation methods to obtain desired kinematic and/or dynamic information. However, due to the structural and functional heterogeneity of muscles and the inherent stochastic nature of EMG signals, methodologies of motion estimation from EMG have been researched with various kinematic or dynamic constraints (Staudenmann et al., 2010; Zhang et al., 2011, 2013; Clancy et al., 2012; Li et al., 2014). Most estimation methods are developed based on various machine learning algorithms, such as linear-regression (LR), non-negative matrix factorization (NMF) and artificial neural network (ANN). In the context of multi-DoF estimation, a time-delayed artificial neural network was applied to predict the shoulder and elbow joint angles with average root-mean-square (RMS) errors of 20° on able-bodied subjects during 30-s arm movements (Au and Kirsch, 2000). By using principle component analysis (PCA) for both the EMG and motion data decomposition, the shoulder and elbow motions were estimated in the low-dimensional space by a state-space decoding model (Artemiadis and Kyriakopoulos, 2010, 2011). Although the estimation accuracy was not explicitly reported, it was sufficient for the tele-operation of a robotic arm in the 3-D Cartesian space (Artemiadis and Kyriakopoulos, 2010). However, the non-stationarity characteristics of the EMG signals were still troublesome (Artemiadis, 2012), so that the decoding model had to be switched in the course of motion completion according to the muscle contraction states of each muscle involved in order to compensate the motion estimation deviation over time in 3-min arm movements (Artemiadis and Kyriakopoulos, 2011).

Apart from the nonlinearity and non-stationarity, subject-specific and crosstalk problems have always companied with the application of EMG whose characteristics apt to be influenced by both physiological and nonphysiological factors during dynamic movements (Beck et al., 2006; Artemiadis, 2012). Thus, it was difficult to find consistent patterns among subjects regarding the motor control strategies (Beck et al., 2006). Even for single subject, since the concentric and eccentric muscle contractions adopted different motor control strategies, the joint angle had different effects on related EMG signals (Qi et al., 2011). Therefore, when shoulder and elbow coordinate to generate large-range arm motions, the EMG-based simultaneous and continuous estimation of the involved joint kinematics is complicated. This paper attempts to simultaneously and continuously estimate three shoulder angles and one elbow angle without switching the motion estimation model, which is validated with up to 2-min arm motions on six subjects.

This work proposes an alternative method to simultaneously and continuously estimate the joint angles from EMG signals during multi-joint coordinated arm movements. With respect to the previous works, EMG-driven estimation of 4 DoFs across 2 joints (shoulder and elbow) is investigated in this work during coordinated arm movements which are involved in daily activities. The estimation performance was evaluated by multiple motion repetitions up to 2 min in order to evaluate the robustness of the proposed methods to time-variant characteristics of EMG. During all the estimation processes, only one estimation model is learned and used for motion estimation for single subject without the model switching during the course of motion completion.

## 2. Methods

The coordinated upper limb movements, such as reaching, pointing, exercising and manipulating, play important roles in human daily life. Although the three main joints of upper limb, shoulder, elbow and wrist, represent distinct kinematic and dynamic properties, their coordination determines the completion of human intended movement. Correspondingly, each muscle activating these joints has different spatial and temporal pattern. Wherein, the shoulder and elbow joints confer the wide range of motion for upper limb, so the coordination of the 3 DoFs at shoulder (flexion/extension, abduction/adduction and pronation/supination) and 1 DoF at elbow (flexion/extension) were investigated in this work with the wrist joint being excluded for simplification as did in Artemiadis and Kyriakopoulos (2010) and Artemiadis and Kyriakopoulos (2011). In our opinion, it is more meaningful to estimate shoulder and elbow DoFs simultaneously and continuously as there are more variabilities during a wide range of their motion comparing with wrist. The pattern recognition-based human-machine interface (HMI) cannot represent such instantaneous variations except static and discrete motion pattern. This study was approved by the local ethical committee of Huazhong University of Science and Technology, and all subjects signed informed consent forms.

### 2.1. Experiment setup

Six able-bodied subjects (all male, 23 ± 1 year-old, 62 ± 4.5 kg) participated in this study. All of them are right-handed dominant and have no reported neuromuscular disorders of their upper limb. They were ever instructed to practise the intended movements until they feel comfortable to the experimental setup.

#### 2.1.1. EMG recordings

**Figure 1:**
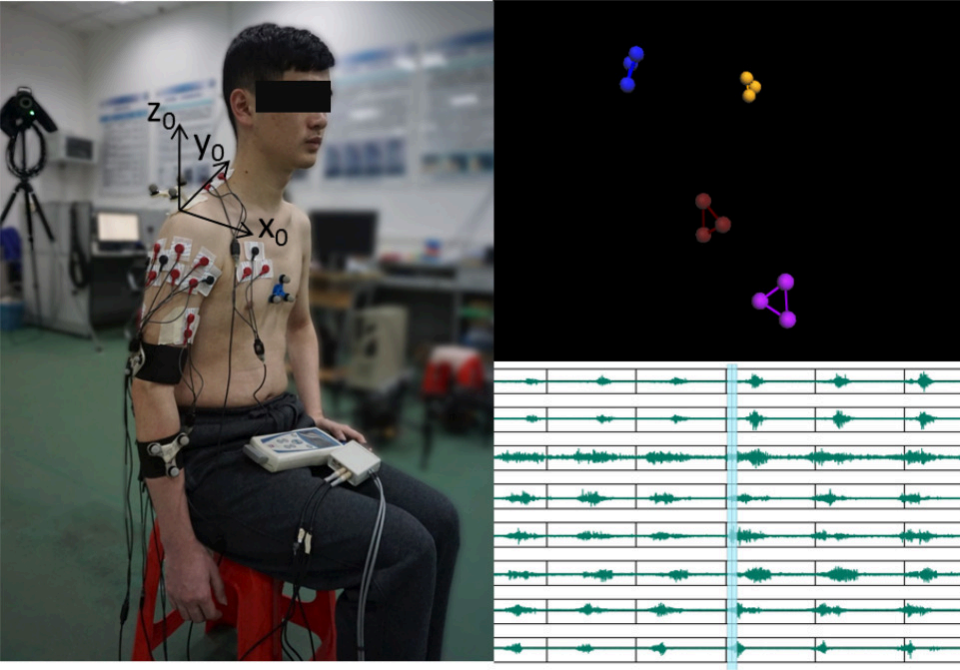
**Illustration of the experiment setup**. The configuration of the EMG electrodes and reflective marker clusters is shown on the left plot with the bony landmarks being removed. Correspondingly, the motion recording marker clusters and EMG traces are respectively shown on the top right and bottom right. (The subject in the present image provided written informed consent for the publication of this image.)

Surface EMG signals were acquired by a commercial EMG acquisition system (Me6000, Mega Electronics Ltd, Kuopio, Finland). The configuration of the EMG recording is shown in Figure 1. Eight predominant muscles activating the four shoulder and elbow DoFs were selected to be the muscle of test, that is, biceps, triceps, deltoid (anterior), pectoralis major (clavicular head), deltoid (middle), deltoid (posterior), trapezius, and teres major muscles. Eight channel of bipolar differential amplifier were carefully placed on these muscles according to both the anatomy and hand touch experience. The active EMG electrodes of each channel were positioned at the muscle belly along the muscle fiber direction with the reference electrode orthogonal to the midline of the active electrodes according to the recommendation of Me6000. The skin underneath the electrodes was cleaned with alcohol patch to reduce the resistance between the skin and the electrodes. The EMG signals were amplified (gain 305) and sampled at 1 KHz.

#### 2.1.2. Motion recordings

A 6-camera motion capture system (Vicon F20-MX3, Vicon Motion Systems Ltd, Oxford, UK) was used to record the motions of the upper limb. The Euler angles were calculated from the reflexible marker positions as proposed previously (Chen et al., 2010). During the dynamic motion recording, only four marker clusters were needed (as shown in Figure 1). The recording marker clusters were attached respectively at the flat part of the acromion, the lateral upper arm just below the insertion of the deltoid, the dorsal surface of the distal forearm. The reference marker cluster was located at the flat part of sternum close to the jugular notch. Before the dynamic motion recording, seven bony landmarks were added to specific positions together with the marker clusters according to the recommendation on definitions of joint coordinate systems (Wu et al., 2005). The position relationship between the bony landmarks and the marker clusters was established in a static recording trial. The position of the bony landmarks could then be reconstructed by the marker clusters and their relationship after the bony landmarks were removed. The advantage of such process, reconstructing the bony landmarks from the rigid marker clusters rather than directly recording the bony landmark positions, is to eliminate the recording errors due to the relative bone movement across specific joints. The motion recording was sampled at 50 Hz and synchronized with the EMG recording through the motion capture system.

### 2.2. Experimental protocol

**Figure 2:**
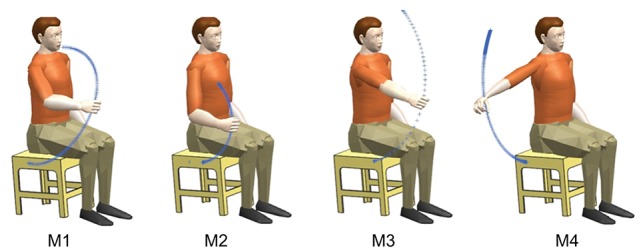
**Illustration of the movements and their trajectories involved in this study**. All these movements were resulted from the coordination of multiple muscles and multiple joints. Each movement was initiated from the lowest position and returned to the initial position after the completion of the movement.

During the experiment, the subjects were quietly seated in the chair with their torso keeping upright and their right hand keeping relaxing. Considering both the functional tasks of upper limb (van Andel et al., 2008) and the present experimental setup (for example, some movements having serious occlusion problems of the reflexive markers during the course of movement have to be excluded), four motions shown in Figure 2 were designed to obtain sufficient range of motion of the right upper limb. All the motions were initiated when the right arm was freely hanging and close to the torso. The traces in Figure 2 indicated the movement trajectory from the initial position to the destination position in a single trip of each motion. Motion M1 simulated delivering water to drink. In motion M2, the subjects were asked to touch their right shoulder with their right hand in the sagittal plane. In motion M3 (M4), the subjects were asked to lift their right arm in the sagittal (coronal) plane as they can. After the completion of each movement, the right arm returned to the initial position. As most daily-life movements of human are quasi-rhythmic after sufficient learning and training except when unexpected events happen (Churchland et al., 2012; Kandel et al., 2012), these four arm movements are freely repeated several times in order to capture both the commonality and the variability of the EMG and motion properties.

**Figure 3:**
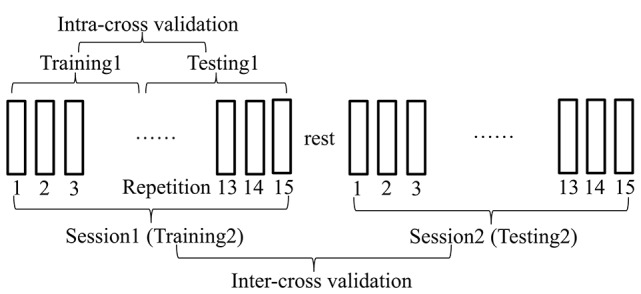
**Description of the experimental protocol**. Two separate motion sessions with each session consisting of 15 repetitions of the motion were designed to collect sufficient experimental data and catch most variabilities during the movements. The muscles were allowed to relax between the adjacent motion repetitions within each test session and between two separate test sessions. When different data were used for training and testing of the estimation procedure, the cross-validation procedure was defined as intra-cross validation (training1, testing1) and inter-cross validation (training2, testing2) respectively.

In order to collect sufficient experimental data and catch most variabilities during the experiments, we designed two separate motion sessions with each session consisting of fifteen repetitions of the motion (see Figure 3) for each motion of each subject. The muscles were allowed to relax shortly (around 3 s) before initiating the next motion repetition. Around 5-min rest was allowed before the start of the second test session. All the motions were performed naturally without any kinematic or dynamic constraints of the right arm. The exact duration of the single motion completion and the rest between adjacent motion repetitions were controlled by the subjects themselves as they felt comfortably. Such experimental setup without strict kinematic constraints was also suitable to verify the feasibility of the proposed joint angle estimation method in practical myoelectric control scenario.

### 2.3. Data processing

With the above EMG and motion recording strategies, both of the EMG and motion data were saved in a computer and treated off-line in Matlab (The MathWorks, Version 7.10.0.499, 64-bit, 2010). The purpose of data processing is to extract suitable signal features, including EMG and motion features, for estimation model learning. In addition, the test data used for cross validation were also processed as did for the training data.

As we know, the muscle synergies and motion synergies contribute to reducing the complexity of neural control in the redundant human motor system. Mathematics techniques, such as PCA, can be used to parse the complex data into a small number of components, for example, only two synergies were needed to account for more than 96% of the activation patterns of eleven human arm muscles during free arm motions (Artemiadis and Kyriakopoulos, 2011). Independent component analysis (ICA) was also effective to extract statistically independent muscle activity source signal from their combinations from multi-channel EMG (Nakamura et al., 2004; Garcia et al., 2005) and high-density EMG signals. ICA has been considered superior to PCA in improving force estimation accuracy from EMG (Staudenmann et al., 2007). Inspired by these previous works, two mode decomposition methods, PCA and ICA, were respectively conducted following band-pass filtering (6th Butterworth with 10–400 Hz cut-off frequency) of the EMG signals.

The multi-channel of EMG recordings are usually noisy and have super-Gaussian or Gaussian distributions in different activation levels (Nazarpour et al., 2013). PCA is selected to process the EMG signals as it is especially efficient in dimension reduction when the signals are highly correlated. With the PCA processing, the recorded EMG signals can be on one hand transformed to the low-dimensional principal components (PCs) with noise free. On the other hand, the dependence among different recording channels can be broken resulting in uncorrelated signals. As for the ICA algorithm, it works under the hypothesis that the original signals are linearly mixed and have non-Gaussian distribution, so that it is reasonable to perform PCA as a preprocessing for ICA to discard the irrelevant structures among the EMG signals and satisfy the hypothesis of ICA algorithm. From now on, whenever we mentioned ICA, it means ICA was conducted after preprocessing of PCA while PCA means only PCA was conducted. The detailed algorithm of PCA and ICA can be found in Johnson and Wichern (2007) and Hyvarinen et al. (2001).

Noth that, the feature vector obtained by the training data with PCA was saved for the testing data to be projected in the same low dimensional space as did on the training data. With ICA, the estimation of unmixing matrix can be obtained through different ways, such as the approximations of negentropy, the minimization of mutual information and the maximum likelihood estimation. We achieved the estimation of unmixing matrix through negative entropy maximization due to its advantages such as fast convergency and robustness (Hyvarinen et al., 2001). Similarly, the unmixing matrix obtained with the training data was kept for the testing data to be unmixed in the same way as did on the training data.

After the PCs (ICs) of the recorded EMG signals were induced from PCA (ICA), the EMG feature set, including mean absolute value (MAV), root mean square, waveform length, zero crossing, variance, median frequency and mean frequency were further calculated in a 40 ms analysis window with 50% overlapping. Although such feature set was applied or partly applied in previous works (Artemiadis and Kyriakopoulos, 2011; Jiang et al., 2012), we did not find consistent improvement in estimation quality when using the combined feature set comparing with using only MAV feature. Therefore, we only present the analysis and results by using only MAV as the EMG feature in the following description.

**Figure 4:**
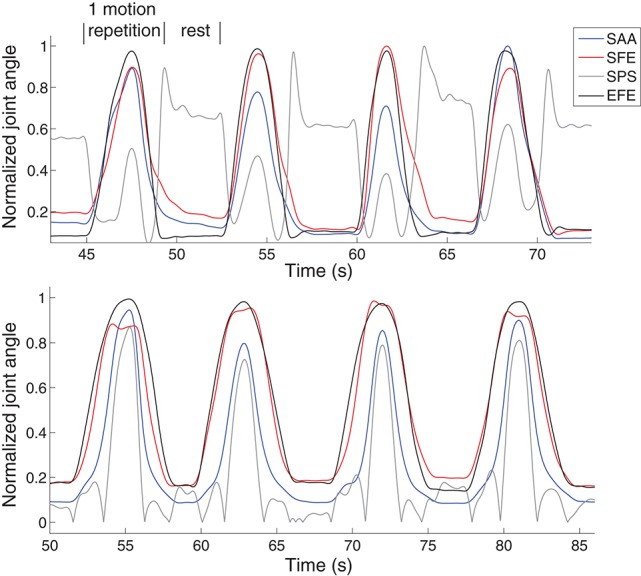
**Examples of 4 motion repetitions of M1 in two subjects**. It is clear to find evidence of redundancy in achieving the same movement with different activating strategies of multiple DoFs in the same subject. The behavior of single DoF and the correlation among all the DoFs are both different in different subjects for the same movement.

For the motion data, 2-order butterworth filter with cutoff frequency of 6 Hz was applied firstly to the recorded reflective marker position data. The three joint angles at shoulder and one joint angle at elbow were then calculated from the marker position data according to the method proposed previously (Chen et al., 2010). The 4 DoFs are defined as shoulder abduction/adduction (SAA), shoulder flexion/extension (SFE), shoulder pronation/supination (SPS) and elbow flexion/extension (EFE). Although the joint motion dimension can be reduced from original 4-fold into 2-fold representing 98% of the total variance as did in Artemiadis and Kyriakopoulos (2010), since we found this processing did not contribute in reducing the complexity or improving the estimation accuracy significantly, the original 4 joint angles were directly used as the estimation target rather than the low-dimensional 2 components. In Figure 4, the 4 DoFs are respectively rectified and normalized by their maximum value to represent in the same range of [0, 1]. As we expect, for the same DoF, it differs over time during different movement repetitions. Among all the DoFs, their correlation differs during different movement repetitions as well, which reveals clear evidence of redundancy in achieving the same movement with different activating strategies of multiple DoFs. In addition, the behavior of single DoF and the correlation among all the DoFs are both different in different subjects for the same movement. Because of these variabilities, simultaneous and continuous estimation of the joint angles is important to interpret instantaneous neural variations for natural and accurate myoelectric control.

Until now, we obtained both the EMG features and joint angle features to be the input and output respectively of the motion estimation model.

### 2.4. Joint angle estimation with ANN

Multi-layer perceptron (MLP), a feedforward ANN model, was used to learn the mapping from the low-dimensional EMG feature to the shoulder and elbow joint angles. The multiple MLPs (mMLPs) has been popularly used for training where one MLP outputs one joint angle in order to obtain accurate estimation for each joint angle (Jiang et al., 2012; Muceli and Farina, 2012). In addition to the separate MLP training for each joint angle estimation, we attempted to use single MLP (sMLP) for concurrent estimation of all the joint angles. The sMLP was then assessed to see if it could be used instead of mMLPs with comparable estimation accuracy and compact training procedure. In each MLP training neural network, there were three hidden layers with five neurons in each layer. The neurons in the hidden layers and output layer had a tangent sigmoid transfer function and a Linear transfer function respectively. The MLP was trained through the Leverberg-Marquardt back-propagation algorithm. All these network training parameters were fixed during the training and testing processes.

**Figure 5:**
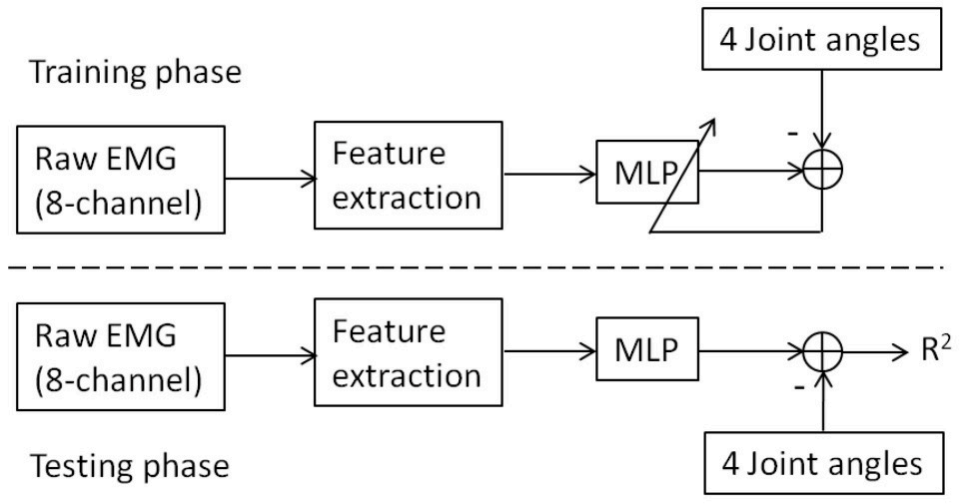
**The illustration of joint angle estimation from EMG through sMLP network**. In the training phase, the MLP were learned by the differences between the measured joint angles and the MLP output. In the testing phase, the trained MLP outputs the joint angle estimates in the presence of new EMG feature data. The performance index *R*^2^ is calculated from the measured and estimated joint angles through Equation (1). In the feature extraction, the raw EMG signals were sequentially processed with band-pass filtering, PCA or ICA decomposition and MAV calculation.

The training and testing of the sMLP strategy are illustrated in Figure 5. In the training phase, the EMG features and joint angles were fed into the MLP, the correlation between them was learned and kept for the following testing phase. In the testing phase, the trained MLP outputs the joint angle estimates in the presence of new EMG feature data. The performance index *R*^2^ is calculated from the measured and estimated joint angles through Equation (1). Note that, in the feature extraction procedure, either PCA or ICA was applied before the calculation of MAV of EMG.

### 2.5. Performance index

The estimation performance can be evaluated by different indices, such as RMS error, correlation coefficient (CC) and coefficient of determination *R*^2^. In order to compare the performance of the proposed method with other works, global *R*^2^ was calculated across all the joint angles as below:

(1) $$\begin{array}{r} R^{2}=1-\frac{\sum_{i=1}^{D}\sum_{t=0}^{N}{\left(\hat{y_{i}\left(t\right)}-y_{i}\left(t\right)\right)}^{2}}{\sum_{i=1}^{D}\sum_{t=0}^{N}{\left(y_{i}\left(t\right)-\bar{y_{i}\left(t\right)}\right)}^{2}} \end{array}$$

where *y*_*i*_(*t*), $\bar{y_{i}\left(t\right)}$ and $\hat{y_{i}\left(t\right)}$ are respectively the real joint angle calculated from motion capture system, its average over time and its estimates from only EMG signals at time instant *t* for the *ith* DoF. *N* is the number of samples of each joint angle. *D* is the number of DoF involved in the study.

All the estimations were performed on the experimental data recorded from six able-bodied subjects. In a word, the human motion estimation process consists of two phases: training and testing. In the traditional estimation method, *k*-fold cross validation (*k* = 4 *or* 5) was popularly applied, where one out of the *k* repetitions of each movement was used as testing set and the rest repetitions as training set (Jiang et al., 2012; Muceli and Farina, 2012). In comparison, we applied leave-*p*-out strategy for the cross-validation where *p* motion repetitions were used for testing with the rest for training. As the variabilities over time likely deviate the estimation result, this strategy is helpful to evaluate the possibility of the trained model in estimating joint angle in continued movement repetitions. If the training and testing data coming from the same test session, we call such traditional cross-validation process as intra-cross validation. In addition, we proposed to conduct inter-cross validation to reveal more convincing results where the training and testing data were coming from two test sessions for each motion in each subject (see Figure 3). Comparing with the intra-cross validation, the inter-cross validation is closer to the practical online condition in myoelectric control, where the testing was performed online following the offline training.

## 3. Results

The purpose of the current work is to propose a simultaneous and continuous joint angle estimation method for multi-joint coordinated upper limb myoelectric control. In the course of joint angle estimation, several factors, such as signal feature extraction method, time-variant properties of EMG and MLP training/testing strategy, may have unexpected influence on the estimation accuracy. Any failure on the estimation course may result in the failure or high error in the joint angle estimation. Therefore, the estimation performance was evaluated in several aspects.

1) ***EMG Mode Decomposition:*** The estimation performance with ICA and PCA decomposition was compared, suggesting their contribution and advantages for the estimation quality. Original 8-channel raw signals were reduced to 3~5-manifold for different subjects representing 95% of the total variances by PCA processing. When the ICA algorithm was conducted, the uncorrelated principal components obtained by PCA was treated as the mixed signals.
2) ***Training Strategy:*** We compared to training strategies in this paper, that is, four joint angles were derived from four MLP networks individually (mMLPs) or from one MLP together (sMLP). Their feasibility and difference were presented and compared by the statistic analysis of the estimation results.
3) ***EMG Non-stationarity:*** As nonstationary EMG property was always a trouble in EMG relevant works, including myoelectric control, the estimation performance of the proposed methods was tested in short-term and middle-term as well, suggesting its feasibility for middle-term use. Either in the intra-cross validation or the inter-cross validation, we divided the testing data into two equivalent parts. The estimation of the first half was called short-term estimation with the last half corresponding to the middle-term estimation.
4) ***Cross-Validation:*** In the intra-cross validation procedure, all the training and testing data were selected from the same test session with half for training and the other half for testing as shown in Figure 3. In this work, around seven motion repetitions were used for testing which can catch more variabilities during the experiments. In the inter-cross validation, the data in session1 were used for training with the data in session2 for testing. In total, there were fifteen motion repetitions being used for testing. The estimation accuracy was the average of the estimates of multiple motion repetitions which was more practical and convincing to represent the overall estimation quality of the proposed methods.

### 3.1. Multi-DoF motion estimation during intra-cross validation

**Figure 6:**
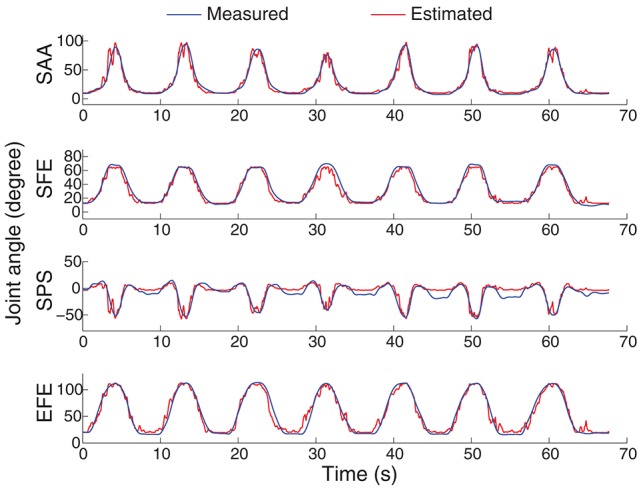
**An example of the 4-DoF estimation on the subject shown at the bottom in Figure 4**. The motion M1 was selected to be shown here. The sMLP network was trained after ICA processing of the EMG signals.

An example of the joint angle estimation during intra-cross validation is presented in Figure 6. The blue lines indicate the joint angles calculated from motion capture system which are considered as the real joint angles. The red lines indicate the joint angle estimates from the EMG and MLP (trained with the training datasets). The estimation was obtained by sMLP and the EMG was processed by ICA. The global estimation accuracy is 94.6% represented by *R*^2^. In such arm movement, multiple muscles and multiple joints were involved to achieve the coordinated muscle contractions and joint movements. Although the motion velocities and the trajectories are both different among different motion repetitions as shown in Figure 4, all the joint angles can be still estimated in the rest, initial concentric and final eccentric motion phase, indicating the feasibility and accuracy of the joint angle estimation method from EMG even in the presence of some kinematic variabilities.

**Figure 7:**
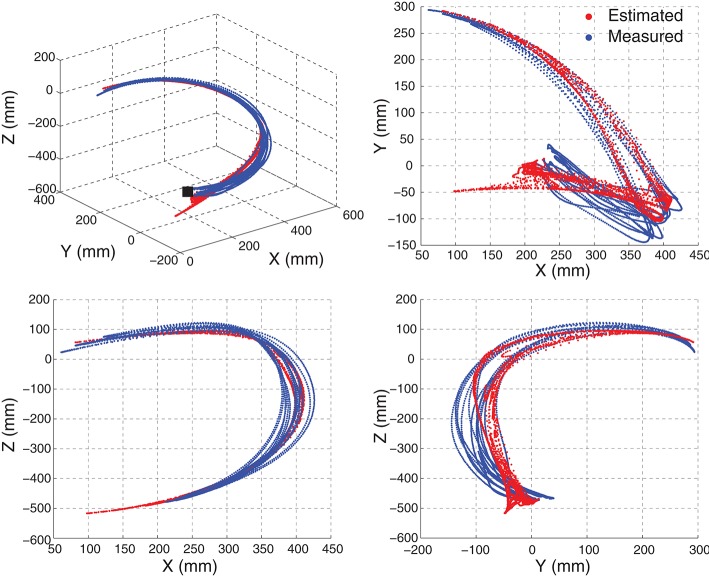
**An example of the movement trajectory of the end-effector constructed by the measured joint angles (blue) and the joint angle estimates (red)**. The motion M1 with seven repetitions, the same as shown in Figure 6, was shown here. The movement trajectory in 3-D space is shown on the left top with the others being the movement trajectory projection in 2-D space. The initiation position of the movement is indicated by a black solid square.

Although the estimates of joint angles can be directly used as the control commands for the robot control, the spacial trajectory of the end-effector is also what we concern. The trajectory must be natural and smooth, otherwise, the joint coordination may be problematic in practice. Therefore, we evaluated the 4 joint angle estimation performance not only by comparing the estimated joint angles with the measured angles, but also by comparing the trajectories of the end-effector driven by the estimated angles and measured angles. The forward kinematics analysis was conducted using the measured and estimated joint angles respectively to compare the measured and estimated trajectories of the end-effector. As the wrist joint was excluded in this work, the center of the wrist joint was considered as the end-effector with the wrist joint being fixed. Figure 7 shows the motion trajectory of the end-effector constructed by the measured joint angles (blue) and the estimated joint angles (red) in Figure 6. In order to show the movement trajectory intuitively, the original coordinate is set at (*x*_0_, *y*_0_, *z*_0_) as shown in Figure 1. In this motion (M1), both the shoulder and elbow joint move in a large range of motion. The determination of *R*^2^ of the trajectory matching is 94.58%. The estimation error mainly exists at the beginning of the movement. Comparatively, the midline and the destination of the trajectory calculated from the estimated joint angles matches the recorded quite well.

#### 3.1.1. Performance comparison with different EMG features and training strategies

**Figure 8:**
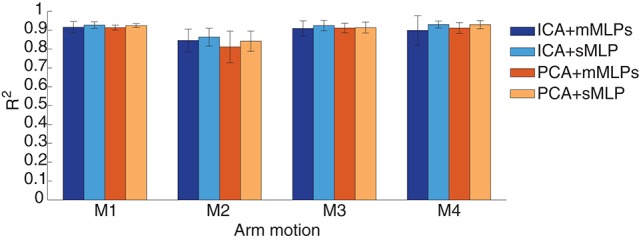
**Performance comparison between different EMG mode decomposition and different MLP training strategies in intra-cross validation**.

PCA and ICA were both considered necessary and useful in EMG mode decomposition and represented their suitability in EMG-based joint angle or force estimation (Staudenmann et al., 2007; Artemiadis and Kyriakopoulos, 2010). We intended to evaluate their contribution to the motion estimation performance. Thus, the motion estimation accuracies were calculated and compared between PCA and ICA processing of the EMG signals in this study. For the neural network training, previous works applied mMLPs to yield one angle from each MLP. To simply the training strategy, we compared the estimation performance with sMLP and mMLPs. Finally, sMLP and mMLPs grouping with PCA and ICA algorithm respectively were used for neural network training. All the estimation accuracies of each motion across all the subjects were averaged and summarized in Figure 8. We find that the sMLP is sufficient to yield comparable even better estimation accuracy with respect to the mMLPs either with PCA or ICA for EMG mode decomposition. In addition, the training complexity and computation time is superior when training by sMLP. For the comparison between ICA and PCA, both of them can give high estimation accuracy for each motion. If we average the estimation accuracies across all the motions, the four combinations, that is, ICA+mMLPs, ICA+sMLP, PCA+mMLPs and PCA+sMLP, have respectively yielded 89.23, 91.12, 88.7, and 90.23% estimation accuracy comparing with the measured joint angles. By conducting two-way ANOVA test between the two factors, EMG decomposition method (ICA/PCA) and training strategy (sMLP/mMLPs), the *p*-values for the ICA/PCA, 0.5919, the sMLP/mMLPs, 0.1409, and the interaction between the two factors, 0.8225, indicating all these combinations get similar motion estimation performance and no significant interactions between the two factors. Considering the potential versatility of the ICA and the simple configuration of the sMLP, in the following study, if we did not explain especially, the EMG was processed by ICA and the sMLP was chosen as the training strategy.

#### 3.1.2. Performance comparison between short-term and middle-term estimation

**Figure 9:**
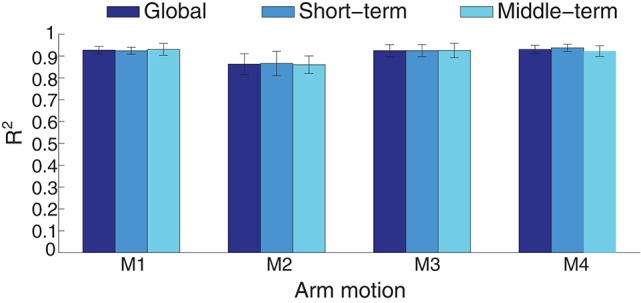
**Performance comparison between short-term and middle-term estimation in intra-cross validation with ICA processing**. The average estimation accuracy is respectively 91.12, 91.29, and 90.97 for the global, short-term and middle-term estimation duration.

Nonstationary properties of EMG is a troublesome effect for myoelectric control. For example, the estimation accuracy decreased from around 30 s without model switching strategy in a free arm movement (Artemiadis and Kyriakopoulos, 2011). The inherent cause may come from the complex electrochemical behaviors of the skeletal muscles. The robustness of the estimation method to the time-related factors was evaluated by comparing the estimation accuracies in the short-term and middle-term application. If any stage of the estimation process, including feature extraction and training, can not overcome the effects of the time-variant factors, the estimation quality may deteriorate over time. In Figure 9, the estimation performance in short-term and middle-term was summarized in the condition of intra-cross validation. The global estimation accuracy is the averaged *R*^2^ when all the test data were used for estimation. When the first half of the testing data were estimated, the short-term accuracy was calculated, whereas the last half of the testing data corresponding to middle-term estimation. The performance across the estimation duration was not statistically significant (*p*=0.9676), indicating the estimation performance is similar during the 70-s arm movements.

### 3.2. Multi-DoF motion estimation during inter-cross validation

**Figure 10:**
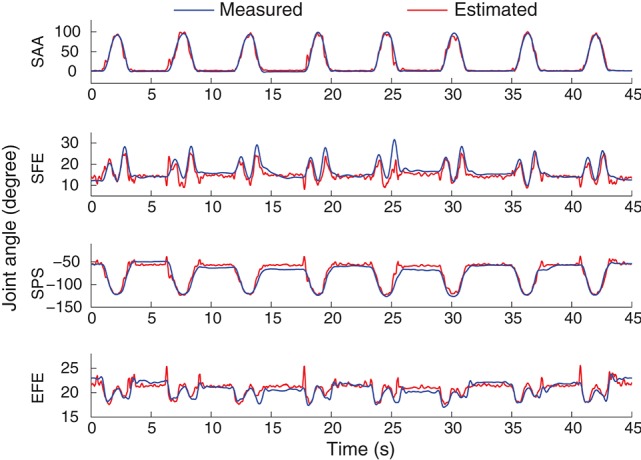
**An example of the 4-DoF estimation result during the inter-cross validation**. The motion M4 was selected to be shown here. The sMLP was trained after ICA processing.

**Figure 11:**
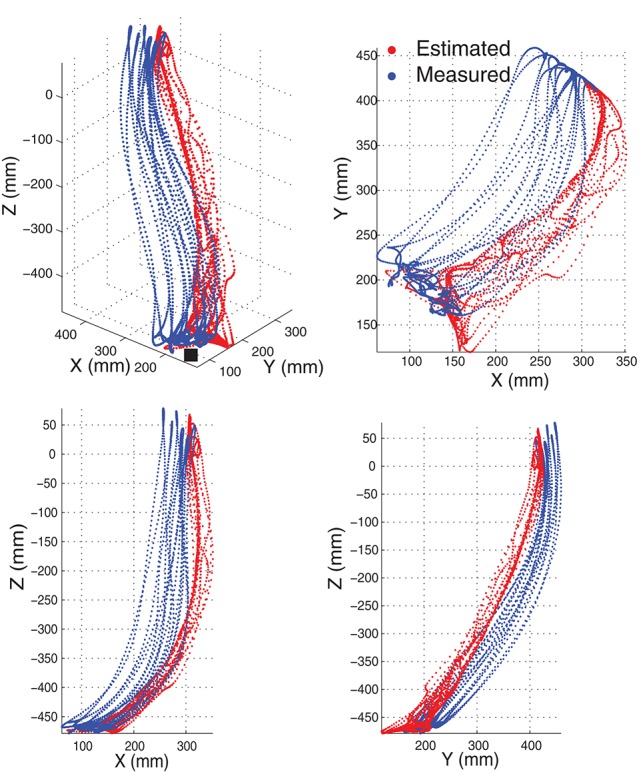
**The movement trajectory of the end-effector constructed by the joint angles in Figure 10**. Motion M4 with seven motion repetitions was estimated and demonstrated. The motion trajectory in 3-D space was shown on the top left. The others were the projection of the trajectory in 2-D space. The initiation position of the movement is indicated by a black solid square.

In intra-cross validation, part of data from the same motion session was used for training with the other part for testing. In practice, when the training phase finished, a new motion session starts and new EMG signals may result in corresponding estimates. Therefore, we also tested the motion estimation using EMG signals from two different sessions for training and testing respectively, which was more practical from the muscle states and estimation conduction procedure. An example of the joint angle estimation during the inter-cross validation is shown in Figure 10. The estimation accuracy is 98.95% for the motion M4 of this subject in the 2-min estimation. In this motion, the subjects were asked to lift their right arm in the coronal plane. Due to no kinematic or dynamic constraints being exerted, the subject could perform this motion as they felt comfortably. For example, they may flex their elbow to compensate the uncomfortability during the motion. In this motion, the 4 joint angles were ranged quite differently, while the joint angle estimates were quite close to the measured angles. The corresponding motion trajectory estimation accuracy is 91.38% (shown in Figure 11). Although the practical initiation positions and trajectories (blue) in different motion repetitions are scattering in each plane, the constructed estimate of the motion trajectories (red) are still smooth and accurate in reaching destination.

**Figure 12:**
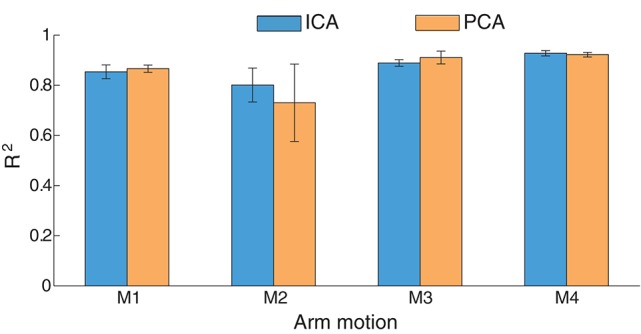
**Statistics results (***R***^**2**^) between ICA and PCA in inter-cross validation**. The results were averaged across six subjects.

**Figure 13:**
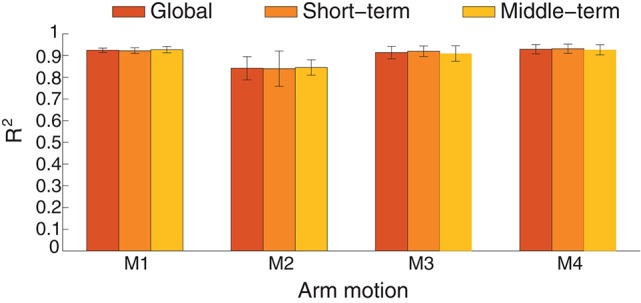
**The joint angle estimation performance is compared between short-term and middle-term estimation in inter-cross validation across six subjects**.

The estimation comparison between ICA and PCA in this inter-cross validation is shown in Figure 12. The average estimation accuracy with ICA and PCA of four motions across six subjects is 87 and 86.3% respectively. The estimation performance seems similar in total with ICA or PCA for EMG mode decomposition in this inter-cross validation (*p* = 0.8531), except for the motion M2 where ICA presents apparent advantages comparing with PCA. With respect to the intra-cross validation where 91.12% (90.23%) estimation accuracies were presented in Figure 8, the estimation in the inter-cross validation is a little worse. However, considering the practical condition of myoelectric control and the estimation accuracies of the previous works as described in the introduction, this estimation performance is quite satisfactory. Moreover, the proposed method also works well during 2-min estimation process in the inter-cross validation. The average estimation accuracy was summarized in Figure 13 during middle-term estimation with ICA. There are also no significant differences among the global, short-term and middle-term estimation performance (*p* = 0.8870).

## 4. Discussion

This paper investigated the feasibility of simultaneous and continuous motion estimation from EMG during arm movements involving coordinated activation of four DoFs across shoulder and elbow joints. In comparison to single joint (such as wrist or elbow joint), the coordination of shoulder and elbow joints offer more contribution for the upper limb to reach objects in a large range of motion. Thus, the simultaneous and continuous estimation of shoulder and elbow motion is important and challenging due to the complex activation of multiple muscles and multiple DoFs (Prilutsky et al., 2011). Although only four arm motions were involved in this work, all of them were functional movements popularly in daily life and achieved by activating all the four DoFs under the condition of no kinematic or dynamic constraints. It is especially meaningful in rehabilitation robotics systems where functional movements are practically important for the users (Takahashi et al., 2008). Moreover, such rehabilitation robot is usually able to provide functional but few movements due to the tradeoff between the complexity of mechanical configuration and the complexity of control system (Maciejasz et al., 2014).

Myoelectric control based on simultaneous and continuous motion estimation has been considered superior to that based on pattern recognition as it provides continuous motion (joint angle) estimates as long as the EMG signals are available. In this work, the proposed method either with PCA or ICA for EMG mode decomposition yields accurate joint angle estimation of the 4DoFs at shoulder and elbow joints. The estimation performance was not only evaluated by intra-cross validation and inter-cross validation, but also evaluated in short-term and middle-term condition. In intra-cross validation, ICA and PCA with sMLP training respectively gives 91.12 and 90.23% estimation accuracy on average over four arm motions across six subjects. In inter-cross validation, the corresponding estimation accuracy is 87.14 and 86.30% respectively. This estimation accuracy is high in the context of simultaneous and continuous estimation of multiple DoFs. In addition, the estimation model was not needed to be switched even during 2-min movement, which is relatively stable and efficient for online myoelectric control.

In this preliminary study, only able-bodied subjects were involved. In this situation, the method can be directly used for tele-operation of assistive robots as in Artemiadis and Kyriakopoulos (2011) and any mechatronics device as long as we would like to control it by our movement intent. This method can be also applied for myoelectrical control of exoskeletons of upper limb during the rehabilitation stage of muscle force enhancement. Furthermore, this method is quite promising to be applied for arm prosthetics control benefiting from more motor information provided by targeted muscle reinnervation (TMR) which has been proved able to use EMG decoding to regain intuitive and simultaneous functional control (Miller et al., 2008; Kuiken et al., 2009; Hargrove et al., 2013). In the proposed method, PCA and ICA both provided satisfactory estimation accuracies in the proposed simultaneous motion estimation, while the latter is a little better due to its capacity in further separating the source muscle activities following muscle synergies extraction in the surface EMG recording scenario. Moreover, ICA is expected to be more advantageous comparing with PCA in the presence of more muscle activity cross-talks through high-density EMG recordings or multiple EMG recordings from the targeted muscles after TMR surgery.

The accurate and stable estimation of multiple DoFs from muscle activities can not only contribute to practical myoelectric control in various robotic systems, but also reveal the inherent correlation between the muscle activities and the coordinated joints through suitable feature extraction and correlation learning methods. Such correlation may be different among different motion tasks, yet determinable for the same motion task even multiple joints and multiple muscles were involved to obtain the coordinated motions.

## 5. Conclusions and perspectives

EMG signals have been popularly used to decode human motion intent for myoelectric control. Traditional motion estimation method uses pattern recognition to provide binary control commands which can only move the robot in a predefined pattern. Simultaneous and continuous motion estimation from EMG has been proposed to provide more natural and accurate motion control command with respect to pattern-recognition based estimation. However, few works have achieved significant progress in simultaneous and continuous estimation of multi-joint coordinated motions. In this work, we proposed a simultaneous and continuous estimation method which can accurately estimate 4 joint angles across two joints for coordinated upper limb motions only from multi-channel EMG signals. EMG mode decomposition through PCA and ICA both revealed their effectivities and feasibilities to obtain accurate joint angle estimation with ICA a litter better. A sMLP network was proposed to estimate the joint angles from the EMG signals after short training. The experimental data from six able-bodied subjects were used to train the neural network and validate the joint angle estimation performance. By comparing the estimated joint angles with the calculated joint angles from the motion capture system, we can find the joint angle estimation accuracy is quite high which indicates that the inherent correlation between the muscle activities and joint angles during complex coordinated motion tasks is feasible to be explored through the proposed mode decomposition and training methods. As the application of EMG decoding in the assistive robot teleoperation (Artemiadis, 2012) and the robotic leg control after a TMR surgery (Kuiken et al., 2009; Hargrove et al., 2013), the accurate arm motion estimation is promising to be applied to control the assistive robot, prosthetic arm or any mechatronics system that we expect to control by our movement intent. In the early future, the proposed method will be further researched on myoelectric control of an exoskeleton our team developed (Liu et al., 2013) and increasing the motion of range of upper limb.

## Author contributions

QZ: Conceived this study. QZ and RL: Performed the experiments and collect the data. WC: Calculated the joint angle from position data. QZ and CX: Proposed the EMG signal processing method. QZ and RL: Analyzed the data and implemented the motion estimation. QZ: Wrote the manuscript. All the co-authors helped revising the manuscript and agreed with the present version.

### Conflict of interest statement

The authors declare that the research was conducted in the absence of any commercial or financial relationships that could be construed as a potential conflict of interest.
